# Causal effect of tooth loss on depression: evidence from a population-wide natural experiment in the USA

**DOI:** 10.1017/S2045796021000287

**Published:** 2021-05-06

**Authors:** Y. Matsuyama, H. Jürges, M. Dewey, S. Listl

**Affiliations:** 1 Department of Global Health Promotion, Tokyo Medical and Dental University, Bunkyo-ku, Japan; 2 Schumpeter School of Business and Economics, University of Wuppertal, Wuppertal, Germany; 3 Department of Health Service & Population Research, King's College London, London, UK; 4 Department of Dentistry – Quality and Safety of Oral Healthcare, Radboud University Medical Center, Radboud Institute for Health Sciences, Nijmegen, The Netherlands

**Keywords:** Chronic conditions, depression, epidemiology, statistics

## Abstract

**Aims:**

Depression severely affects people's health and well-being. Oral diseases have been suggested to be associated with depression, but so far, there is no causal evidence. This study aimed to identify the causal effect of tooth loss on depression among US adults in a natural experiment study.

**Methods:**

Instrumental variable analysis was conducted using data from 169 061 respondents born in 1940–1978 who participated in the 2006, 2008 or 2010 waves of the Behavioral Risk Factor Surveillance System (BRFSS). Random variation in tooth loss due to differential childhood exposure to drinking water fluoride was exploited as an instrument.

**Results:**

US adults who were exposed to drinking water fluoride in childhood had more remaining teeth, therefore providing a robust instrument (*F* = 73.4). For each additional tooth loss, depressive symptoms according to the eight-item Patient Health Questionnaire depression (PHQ-8) score increased by 0.146 (95% CI 0.008–0.284), and the probability of having clinical depression (PHQ ⩾10) increased by 0.81 percentage points (95% CI −0.12 to 1.73).

**Conclusions:**

Tooth loss causally increased depression among US adults. Losing ten or more teeth had an impact comparable to adults with major depressive disorder not receiving antidepressant drugs.

## Introduction

Major depression is among the leading causes of burden of disease worldwide (GBD 2017 Disease and Injury Incidence and Prevalence Collaborators, 2017). It is not only one of the most common and most detrimental mental disorders affecting psychosocial functioning but also known as a risk factor for various other outcomes such as dementia (Kaup *et al*., 2016). The lifetime prevalence of depression was estimated at 6–21% (Bromet *et al*., 2011), implying that one in five people experience depression at some point in their life. The years lived with disability attributed to depression has increased, accounting for 8% of global health loss in 2010 (Vos *et al*., 2012).

Oral diseases are another major public health issue affecting more than 3.5 billion people worldwide and imply the third highest treatment costs after diabetes and cardiovascular diseases (Listl *et al*., 2015; Peres *et al*., 2019). Despite its critical relevance for people's quality of life and well-being, the prevention and management of oral diseases has been subject to woeful neglect and significant policy failures (Watt *et al*., 2019). One of the main reasons for such neglect could be the previous absence of causal evidence for links between oral health and other primary relevant health outcomes, particularly mental health. Oral conditions have previously been reported to be associated with people's mental health and well-being but, so far, there is no causal evidence for links between oral conditions and mental health outcomes (Cademartori *et al*., 2018). Deciphering the mechanisms between oral and mental health outcomes is not only relevant to better target prevention and treatment of these conditions but also critical for global health advocacy to adequately prioritise mental and oral health on the global health policy agenda. Potential causal links between oral and mental health conditions must be critically scrutinised.

More specifically, there is no causal evidence on the impact of teeth on depression. It has been hypothesised that neuroinflammation due to past periodontal inflammation or autonomic nerve imbalance due to oral-related pain, stress and discomfort increases depressive symptoms; thereby, tooth loss is expected to increase depression (Cademartori *et al*., 2018). Declines in social functioning due to poor oral health (Rouxel *et al*., 2017) might also explain such associations.

The main shortcomings in previous literature about the effect of oral conditions on depression relate to the possibility of reverse causation or other confounding bias. The instrumental variable (IV) approach is a quasi-experimental method to estimate causal effects in observational data. It is a potentially powerful tool, especially when a randomised controlled trial (RCT) is not feasible due to ethical concerns or practical difficulties (Angrist and Krueger, 2001; Maciejewski and Brookhart, 2019). Studies on the association between oral health and depression are typical cases, as both conditions take a long course and are thus difficult to be followed up. The IV approach emulates an RCT by employing exogenous, quasi-randomly assigned variation in a so-called IV – or instrument – that drives the probability or intensity of an exposure or treatment. Under the assumption that the IV has no direct effect on the outcome, the IV method identifies a causal effect even in the presence of unobserved confounders.

Water fluoridation is an evidence-based, robust measure for caries prevention (McDonagh *et al*., 2000), and the benefit continues for tooth loss in adulthood (Neidell *et al*., 2010). Adverse effects of water fluoridation on other outcomes such as cognitive ability have not been observed in many reviews (McDonagh *et al*., 2000). Accordingly, fluoride utilisation is a candidate instrument to examine the causal effect of tooth loss on depression. In the USA, water fluoridation has been implemented since 1945, and the year of introduction and the proportion of people supplied fluoridated water varied by county (eFig. 1 in the Supplementary Material) (US Department of Health and Human Services, 1993). This natural experiment induced historical and geographical variation in fluoride exposure in the US population. By employing this exogenous difference, we scrutinised the causal effect of tooth loss on depression in US adults.

## Method

### Data

The data of the cross-sectional survey of the Behavioral Risk Factor Surveillance System (BRFSS) waves 2006, 2008 and 2010 were pooled and analysed. The BRFSS is a state-based telephone survey of the population aged 18 years or older in the USA (Centers for Disease Control and Prevention, 2010). The median state response rate was 51.4, 53.3 and 54.6% in 2006, 2008 and 2010, respectively (Centers for Disease Control and Prevention, 2010). We used all waves and states that contain information on the number of lost teeth, depression and county of residence. The analytical sample was restricted to respondents born in 1940–1978 because information on water fluoridation during youth was available only for these cohorts. Further excluding the respondents with missing data, 169 061 respondents’ data were included in the analysis (eFig. 2 in the Supplementary Material). This study was approved by the ethical committee at Tokyo Medical and Dental University. This study followed the Strengthening the Reporting of Observational Studies in Epidemiology (STROBE) reporting guideline.

### Instrumental variable

Our instrument was the exogenous variation in water fluoridation by county in the USA, namely the geographical and historical difference in the proportion of people supplied with fluoridated water. County-level water fluoridation is a suitable instrument because it affects the number of lost teeth in the residents through its preventive effect on dental caries (McDonagh *et al*., 2000), while it does not influence the incidence of depression directly and it is arguably not correlated with unobserved determinants of depression.

The information on water fluoridation was obtained from the 1992 Water Fluoridation Census (US Department of Health and Human Services, 1993), which reported the number of the population supplied with fluoridated water by counties every year from 1945 to 1992. We followed a previous study (Glied and Neidell, 2010) and accumulated the county-level proportion of people supplied fluoridated water across years when respondents were 5–14 years old, which corresponds to post-eruptive enamel maturation of permanent teeth (i.e. sensitive for benefit from fluoride). The total population was fixed to the 1990s estimate. The variable was rescaled between 0 and 1 in the analysis so that the coefficient indicates the difference between no exposure and full exposure of water fluoride.

### Treatment variable

The treatment variable, the number of lost teeth, was assessed by a single question. ‘*How many of your permanent teeth have been removed because of tooth decay or gum disease? Include teeth lost to infection, but do not include teeth lost for other reasons, such as injury or orthodontics*.’ with response options of ‘*None*’, ‘*1 to 5*’, ‘*6 or more but not all*’ and ‘*All*’. We did not consider wisdom teeth and assigned the mid-point for each category (i.e. 0, 3, 16.5 and 28, respectively) to construct a continuous variable for the number of lost teeth in the main analysis. Analysis with a dichotomised variable (⩾1 tooth lost *v*. full dentition; and lost all teeth *v*. having ⩾1 tooth) was also performed.

### Outcome variable

The outcome, depression, was assessed by the eight-item version of the Patient Health Questionnaire (PHQ-8) (Kroenke *et al*., 2001). It consists of eight questions on the frequency that the respondent experienced particular depressive symptoms over the past 2 weeks. In the BRFSS, participants were asked the number of days that they experienced the eight symptoms. The responses were converted to a score between ‘not at all’ (0 points) and ‘nearly every day’ (3 points) (see eMethod 1) (Kroenke *et al*., 2001). The total score (range 0–24) as a continuous variable and a binary variable indicating probable major depression (PHQ-8 ⩾ 10) (Wu *et al*., 2019) were used as outcome variables.

### Analysis

We used a two-stage least squares (2SLS) regression analysis to identify the causal effect of tooth loss on depression. The first stage was a regression of the treatment variable (number of lost teeth) on the instrument (fluoride exposure), adjusting for year of birth, wave of the survey, gender and state of residence. In the second-stage regression, the outcome (depression) was regressed on the predicted number of lost teeth adjusting for the same covariates. Thereby, the second-stage coefficient indicates the causal effect of tooth loss on depression. Ordinary Least Squares (OLS) estimation was also performed to compare the effect sizes because the 2SLS estimate can lead to a greater bias than OLS when the instrument is only weakly correlated with the treatment variable (Angrist and Krueger, 2001).

To evaluate whether the effect of tooth loss varies by population characteristics, analyses stratified by age, year of birth, gender, median household income and dental care utilisation were performed. We also performed a sensitivity analysis by assigning mean, median and mode of the clinically examined number of lost teeth reported in a previous study (Sekundo *et al*., 2019) for the brackets of self-reported 1–5 or 6–27 lost teeth.

## Results

Table 1 describes demographic characteristics of the 169 061 respondents (mean age = 50.0; range of age = 28–70; male = 38.7%). The average number of lost teeth was 4.5 when assigning the mid-point to each category. People losing more teeth reported higher depression symptoms while less likely to have been exposed to fluoridated water in childhood.

**Table 1.** Demographic characteristics of respondents; *N* = 169 061^a^

	The number of lost teeth							
	0 (coded 0)		1–5 (coded 3)		6–27 (coded 16.5)		28 (coded 28)	
*N*	84 347		54 866		20 866		8982	
	*n*/mean	%/s.d.	*n*/mean	%/s.d.	*n*/mean	%/s.d.	*n*/mean	%/s.d.
Total score of PHQ-8	2.74	3.71	3.57	4.50	5.19	5.66	5.48	5.95
Possible major depression (PHQ-8 ⩾ 10)								
No	79 391	94.1%	49 379	90.0%	16 808	80.6%	7053	78.5%
Yes	4956	5.9%	5487	10.0%	4058	19.4%	1929	21.5%
Fluoride exposure in childhood^b^	3.31	3.56	2.81	3.37	2.36	3.13	2.02	2.89
Age at survey	46.82	10.55	51.33	10.22	55.56	8.88	58.35	7.94
Gender								
Men	32 360	38.4%	22 054	40.2%	7778	37.3%	3262	36.3%
Women	51 987	61.6%	32 812	59.8%	13 088	62.7%	5720	63.7%
Wave of survey								
2006	50 066	59.4%	29 646	54.0%	10 857	52.0%	4383	48.8%
2008	13 550	16.1%	9327	17.0%	3332	16.0%	1452	16.2%
2010	20 731	24.6%	15 893	29.0%	6677	32.0%	3147	35.0%

PHQ-8, the eight-item Patient Health Questionnaire depression scale; s.d., standard deviation.

a 36 states were included: Alabama, Arizona, Arkansas, California, Delaware, District of Columbia, Florida, Georgia, Hawaii, Idaho, Illinois, Indiana, Iowa, Louisiana, Maine, Michigan, Minnesota, Mississippi, Missouri, Montana, Nevada, New Hampshire, New Mexico, North Dakota, Oklahoma, Oregon, Rhode Island, South Carolina, Tennessee, Texas, Utah, Vermont, Virginia, West Virginia, Wisconsin and Wyoming.

b Sum of the proportion of people supplied with fluoridated water in the county of residence during the age of 5–14 years.

Table 2 shows the causal effect of the number of lost teeth on depression symptoms (i.e. total score of PHQ-8). The first-stage regression showed that exposure to fluoridated water prevented losing 0.564 teeth [95% confidence interval (CI) 0.435–0.693; *F*-statistic: 73.4]. The reduced form equation showed a negative overall effect of being exposed to fluoridated water on depression symptoms. Under the assumption that fluoride exposure affects depression only via dental health, the second-stage regression showed a causal effect of tooth loss on depression: losing a tooth increased depression symptom by 0.146 PHQ-8 total score points (95% CI 0.008–0.284). The effect size was close to the OLS estimate (coefficient = 0.134; 95% CI 0.131–0.136). When tooth loss was dichotomised, having lost ⩾1 tooth and having lost all teeth also increased depressive symptoms [coefficients (95% CI) are 3.141 (0.100–6.182) and 7.251 (0.053–14.449), respectively].

**Table 2.** Causal effect of tooth loss on a total score of PHQ-8 (ranges 0–24); *N* = 169 061

	Coef.	95% CI	*p*-Value	*F*-statistic
***Treatment variable: number of lost teeth***				
OLS estimation				
The number of lost teeth (continuous)	0.134	0.131–0.136	<0.001	–
2SLS estimation				
Second stage				
The number of lost teeth (continuous)	0.146	0.008–0.284	0.039	–
First stage				
Drinking water fluoride^a^	−0.564	−0.693 to −0.435	<0.001	73.4
Reduced form				
Drinking water fluoride^a^	−0.082	−0.162 to −0.002	0.044	–
***Treatment variable: having lost* ⩾*1 tooth***				
OLS estimation				
Having lost ⩾1 tooth (ref. full dentition)	1.643	1.600 to 1.687	<0.001	–
2SLS estimation				
Second stage				
Having lost ⩾1 tooth (ref. full dentition)	3.141	0.100 to 6.182	0.043	–
First stage				
Drinking water fluoride^a^	−0.026	−0.035 to −0.018	<0.001	36.1
Reduced form				
Drinking water fluoride^a^	−0.082	−0.162 to −0.002	0.044	–
***Treatment variable: having lost all tooth***				
OLS estimation				
Having lost all teeth (ref. having ⩾1 tooth)	2.334	2.238 to 2.430	<0.001	–
2SLS estimation				
Second stage				
Having lost all teeth (ref. having ⩾1 tooth)	7.251	0.053 to 14.449	0.048	–
First stage				
Drinking water fluoride^a^	−0.011	−0.015 to −0.007	<0.001	31.7
Reduced form				
Drinking water fluoride^a^	−0.082	−0.162 to −0.002	0.044	–

CI, confidence interval; OLS, Ordinary Least Squares; PHQ-8, the eight-item Patient Health Questionnaire depression scale; 2SLS, two-stage least-squares.

Adjusted for year of birth, wave of survey, gender and state of residence.

a Sum of the proportion of people supplied with fluoridated water in the county of residence during the age of 5–14 years (rescaled between 0 and 1).

Table 3 shows the effect of the number of lost teeth on probable major depression (i.e. PHQ-8 ⩾ 10). The second-stage regression showed that the probability of major depression increased by 0.81 percentage points (95% CI −0.12 to 1.73) with an additional loss of one tooth. The effect size was slightly larger than the OLS estimate (coefficient = 0.73 percentage points; 95% CI 0.71–0.75). When tooth loss was dichotomised, the probability of major depression increased by tooth loss [coefficients (95% CI) are 17.39 (−2.89 to 37.66) and 40.13 (−7.32 to 87.59) percentage points for having lost ⩾1 tooth and having lost all teeth, respectively].

**Table 3.** Causal effect of tooth loss on possible major depression (PHQ-8 ⩾ 10); *N* = 169 061

	Coef.	95% CI	*p*-Value	*F*-statistic
***Treatment variable: number of lost teeth***				
OLS estimation				
The number of lost teeth (continuous)	0.0073	0.0071–0.0075	<0.001	–
2SLS estimation				
Second stage				
The number of lost teeth (continuous)	0.0081	−0.0012 to 0.0173	0.087	–
First stage				
Drinking water fluoride^a^	−0.5640	−0.6931 to −0.4350	<0.001	73.4
Reduced form				
Drinking water fluoride^a^	−0.0046	−0.0099 to 0.0008	0.092	–
***Treatment variable: having lost* ⩾*1 tooth***				
OLS estimation				
Having lost ⩾1 tooth (ref. full dentition)	0.0840	0.0811–0.0870	<0.001	–
2SLS estimation				
Second stage				
Having lost ⩾1 tooth (ref. full dentition)	0.1739	−0.0289 to 0.3766	0.093	–
First stage				
Drinking water fluoride^a^	−0.0262	−0.0348 to −0.0177	<0.001	36.1
Reduced form				
Drinking water fluoride^a^	−0.0046	−0.0099 to 0.0008	0.092	–
***Treatment variable: having lost all tooth***				
OLS estimation				
Having lost all teeth (ref. having ⩾1 tooth)	0.1308	0.1245 to 0.1372	<0.001	–
2SLS estimation				
Second stage				
Having lost all teeth (ref. having ⩾1 tooth)	0.4013	−0.0732 to 0.8759	0.097	–
First stage				
Drinking water fluoride^a^	−0.0114	−0.0153 to −0.0074	<0.001	31.7
Reduced form				
Drinking water fluoride^a^	−0.0046	−0.0099 to 0.0008	0.092	–

CI, confidence interval; OLS, Ordinary Least Squares; PHQ-8, the eight-item Patient Health Questionnaire depression scale; 2SLS, two-stage least-squares.

Adjusted for year of birth, wave of survey, gender and state of residence.

^a^Sum of the proportion of people supplied with fluoridated water in the county of residence during the age of 5–14 years (rescaled between 0 and 1).

**Fig. 1.**
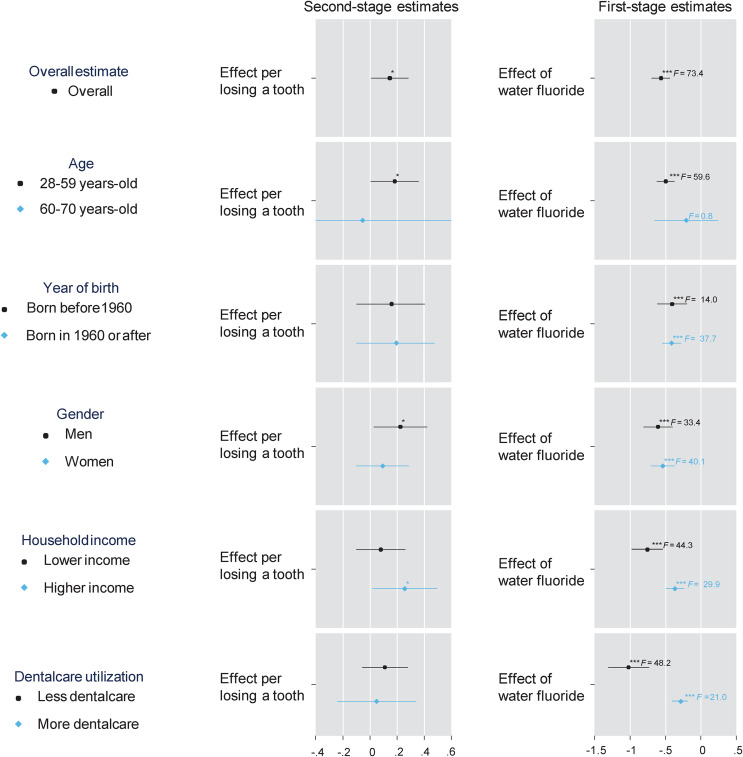
Causal effect of tooth loss on a total score of the eight-item Patient Health Questionnaire depression scale (PHQ-8) with various stratifications; adjusted for year of birth, wave of survey, gender and state of residence; annual household income was divided at the median (<$50 000 or ⩾$50 000); dental care utilization was divided by having visited dentist within one year or not); the left and right panels illustrate the second- and first-stage estimates, respectively; *p < 0.05, **p < 0.01, ***p < 0.001.

The results from the stratified analyses are illustrated in Fig. 1. The effect of tooth loss on depression symptoms appeared to be more pronounced in younger age groups than in older people [coefficients (95% CI) are 0.182 (0.004–0.361) and−0.055 (−1.019 to 0.909), respectively]. The effect sizes did not differ by year of birth. The point estimate for the effect of tooth loss was larger in men than in women [coefficients (95% CI) are 0.225 (0.027–0.422), 0.094 (−0.100 to 0.287), respectively]. When stratified by annual household income, the preventive effect of fluoridated water on teeth was larger for lower-income groups. The point estimate for the effect of tooth loss was larger in the higher-income group than in the lower-income group [coefficients (95% CI) are 0.257 (0.018–0.496) and 0.080 (−0.102 to 0.262), respectively]. The preventive effect of fluoride exposure on teeth was larger among people with less dental care utilisation. The point estimate for the effect of tooth loss was slightly larger in people less using dental care than those who visit dental clinics more [coefficients (95% CI) are 0.110 (−0.058 to 0.279) and 0.049 (−0.242 to 0.341), respectively].

The sensitivity analysis varying the values assigned to the category of tooth loss did not largely change the results, although the second-stage estimate was larger when smaller numbers of lost teeth were assigned (eFig. 3 in the Supplementary Material).

## Discussion

To our knowledge, the present study is the first to identify causal effects of tooth loss on depression, whereby reverse causation and the bias due to potential confounding were eliminated by employing an IV approach. An additional loss of one tooth causally increased depression symptoms by 0.146 points or increased the probability of major depression by 0.81 percentage points. For further interpretation of our findings, we compared our estimates with results from RCTs on antidepressant drugs (Cipriani *et al*., 2018). The effect size of losing ⩾10 teeth was comparable to adults with major depressive disorder not receiving antidepressant drugs (see eMethod 2).

The effect of teeth on depression seemed to be greater in young adults, men, people with higher income and those with less dental care utilisation. Older people may consider tooth loss as a natural change with ageing and have adapted to it (MacEntee *et al*., 1997), thus the psychological impact of tooth loss might be weaker for them. The gender difference is in contrast to women perceiving oral health to be generally more relevant than men (Mason *et al*., 2006). In the USA, the prevalence of edentulousness is higher in women than men across most age groups (Slade *et al*., 2014), suggesting that women are more likely to lose their teeth early. Hence women might be on a different tooth loss trajectory and have adapted to fewer teeth at earlier life stages than men. This might potentially explain some differences in the observed effects of tooth loss for women and men of similar age.

The first-stage regressions stratified by income confirmed the previous literature showing larger benefits of water fluoridation for marginalised people (Riley *et al*., 1999). Similar to previous literature on the marginal return to general health (Grossman, 1972), the larger effect size in the second-stage regressions for the higher-income group might reflect a larger marginal return to oral health for high-income groups. If dental care can partially mitigate the deteriorative effects of tooth loss on depression, a limited affordability of dental care would not only be detrimental to oral health but also exacerbate the suffering from depression in low-income groups.

Depression affects more than 320 million people, or 4.4% of the global population (World Health Organization, 2017); while 2.3% of the global population are edentate (Kassebaum *et al*., 2014). If we could prevent one tooth loss on average, the expected reduction in depression (0.81 percentage points) would account for 60 million people worldwide. We acknowledge this extrapolation might be an oversimplification; however, the present study supports that promoting oral health significantly improves psychological well-being of the global population. Importantly, we identified causal effects from observational data using quasi-randomisation; therefore, the estimated impact on health is not biased by omitted variables or reverse causation. The IV analysis estimates the local average treatment effect (LATE), i.e. effect among compliers (Angrist and Krueger, 2001). In the present study's case, compliers are people prevented from tooth loss by water fluoridation. Water fluoridation is beneficial for all people but has a larger effect on people with a high risk of dental caries (Riley *et al*., 1999).

Most of the previous studies on the effect of tooth loss on depression were cross-sectional analyses. One exception is the study by Yamamoto *et al*. (2017), who followed older people in Japan for 3 years and found that being edentulous was a risk for developing depressive symptoms; however, some potential confounders, e.g. childhood environment, have not been adjusted for. Our approach, IV analysis, can consider all unmeasured confounders between tooth loss and depression. An IV approach can also mitigate the bias due to classical measurement error in the treatment variable (Angrist and Krueger, 2001). As we relied on the government-reported information on water fluoride, respondents’ year of birth and county of residence when deriving the variation in the treatment variable, measurement error might have been mitigated compared to plain self-reported number of lost teeth.

Chronic stress and discomfort lead to hypothalamic-pituitary-adrenal (HPA) axis hyperactivity via dysfunction in the negative feedback system (Malhi and Mann, 2018). The accelerated HPA prolongs cortisol secretion, which is a major biological risk factor for depression (Malhi and Mann, 2018). Among the eight items of the PHQ-8, neurovegetative problems such as sleep problems, having little energy and less appetite/overeating were affected by tooth loss (eFig. 4 in the Supplementary Material). Another mechanism is explained by inflammation due to past or current periodontal diseases because inflammatory cytokines increase depressive symptoms (Malhi and Mann, 2018), although it is less likely in our estimate employing water fluoride as an instrument. Further, a decline in social interaction due to poor oral condition (Rouxel *et al*., 2017) can also explain the result. People with fewer teeth might be less likely to communicate with others because of the problem of eating, speaking or smiling; and that might result in poor mental health outcomes.

The present study has limitations. We used three waves of repeated cross-sectional data, which is limited in terms of temporality between the treatment variable (tooth loss) and the outcome (depression); however, the instrument (exposure to drinking water fluoride in childhood) should be prior to losing teeth. We did not use survey weights although the proportion of gender in the BRFSS survey respondents was lopsided (Centers for Disease Control and Prevention, 2010); it is expected that the effect of tooth loss on depression will increase with the survey weights because the effect was larger in men than in women. It must be noted that we were not able to consider relocation, because only the current county of residence was available. We believe that this induces non-systematic misclassification because it is less likely that people select the county to live to receive the benefit of water fluoridation. In the USA, fluoridated toothpaste was rapidly utilised from the 1970s (Burt and Eklund, 2005). We considered the availability of fluoridated toothpaste by stratifying birth cohorts, and the results were similar. Many studies have shown that water fluoridation is still effective once other fluoride sources are available (Marinho *et al*., 2004). We examined the correlation between county-level characteristics and the proportion of water fluoridation (eTable 1 in the Supplementary Material). Population size and income per capita showed moderate correlation (i.e. spearman's *ρ* >0.20), but adjusting for these variables did not change our findings. A further limitation is related to the self-reported outcome; however, the measurement has been well validated in clinical care settings (Kroenke *et al*., 2001; Sekundo *et al*., 2019). Finally, the findings may not be directly transferrable to other countries or settings, although the prevalence of severe tooth loss in the USA is similar to the global population (Kassebaum *et al*., 2014).

In conclusion, we found sizeable causal effects of tooth loss on psychological well-being. This provides unique and novel evidence underpinning the urgency for taking better action to promote oral health. Further research to identify the causal effects of oral conditions on other aspects of population well-being is strongly encouraged.

## Supplementary material

For supplementary material accompanying this paper visit http://dx.doi.org/ 10.1017/S2045796021000287. click here to view supplementary material

## Data

Data are available from the BRFSS website (https://www.cdc.gov/brfss/index.html).
