# Supplementary material for: Causal effect of tooth loss on depression: evidence from a population-wide natural experiment in the USA
Source: Epidemiol Psychiatr Sci. 2021 May 6;30:e38. doi: 10.1017/S2045796021000287 (PMC8157508; doi:10.1017/S2045796021000287)
Supplement: Supplementary file 1 [file S2045796021000287sup001.docx]

**Online-only supplements**

**eTable 1.** Correlation between the coverage of fluoridated water and county characteristics in 1960, 1970, 1980, and 1990

**eFigure 1.** Fluoridation map in the US; population served fluoridated water in the years of 1950, 1960, 1970, 1980, and 1990 (source: 1992 Fluoridation Census) divided by total population estimates at 1990 (source: Census of Population and Housing)

**eFigure 2.** Flowchart of respondents for analysis

**eFigure 3.** Causal effect of tooth loss on a total score of PHQ-8

**eFigure 4.** Causal effect of tooth loss on each component of PHQ-8

**eMethod 1.** PHQ-8 questionnaire in the Behavioral Risk Factor Surveillance System

**eMethod 2.** Comparison of the effect size with previous randomized controlled trials (RCTs) on antidepressant drugs

**eTable 1.** Correlation between the coverage of fluoridated water and county characteristics in 1960, 1970, 1980, and 1990

|  | Correlation between the coverage of fluoridated water and county characteristics | | | |
| --- | --- | --- | --- | --- |
|  | In 1960 | In 1970 | In 1980 | In 1990 |
|  | ρ | ρ | ρ | ρ |
| County characteristics ^a^ |  |  |  |  |
| Population | 0.19 | 0.27 | 0.24 | 0.22 |
| Median age | - | - | -0.11 | -0.11 |
| Proportion of people with bachelor's degree or higher | - | - | 0.06 | 0.04 |
| Income per capita | 0.21 | 0.19 | 0.11 | 0.07 |
| County's general revenue per capita | - | - | -0.03 | -0.06 |
| County's expenditures for education per capita | - | - | -0.06 | -0.15 |
| County's expenditures for public welfare per capita | - | - | -0.03 | -0.07 |
| County's expenditures for health per capita | - | - | - | -0.06 |
| Births per capita | - | 0.08 | 0.03 | 0.08 |
| Deaths per capita | - | -0.14 | -0.07 | -0.01 |
| Medical clinic per capita | - | - | -0.15 | 0.07 |
| Dental clinic per capita | - | - | -0.14 | 0.11 |

Abbreviation: Spearman's rho, ρ

a data source: U.S. Census Bureau. USA counties: 2011; nearest data before the years of 1960, 1970, 1980, and 1990 were used.

**eFigure 1.** Fluoridation map in the US; population served fluoridated water in the years of 1950, 1960, 1970, 1980, and 1990 (source: 1992 Fluoridation Census) divided by total population estimates at 1990 (source: Census of Population and Housing)


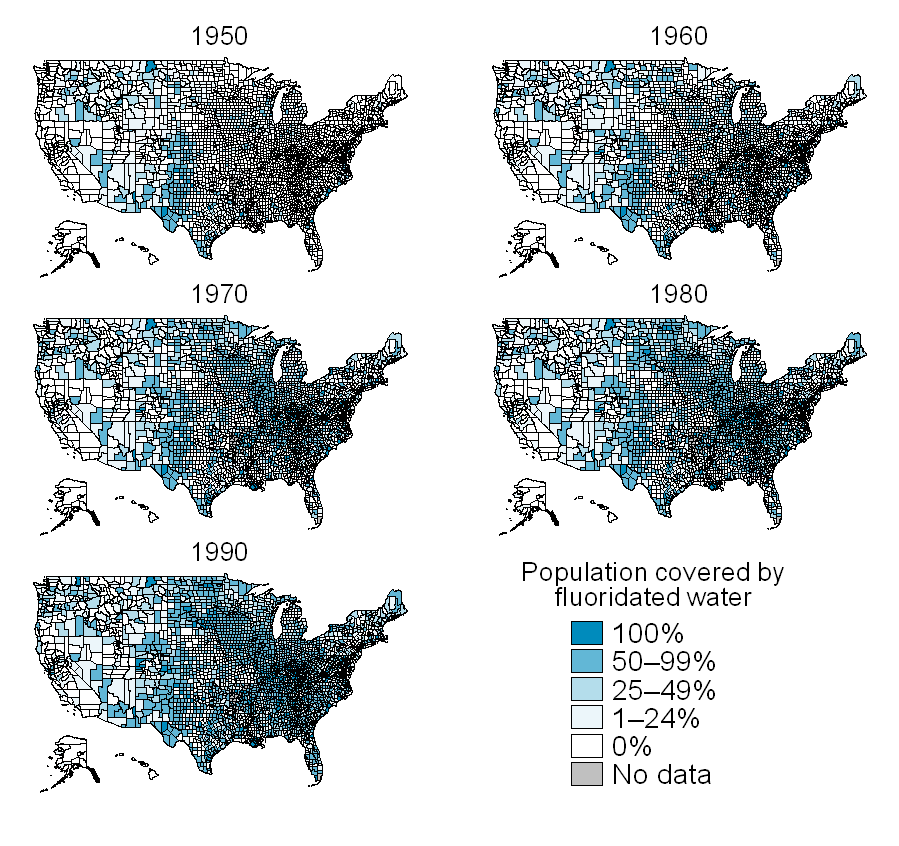


**eFigure 2.** Flowchart of respondents for analysis; BRFSS, Behavioral Risk Factor Surveillance System; PHQ-8, the eight-item Patient Health Questionnaire depression scale

Respondents with information on county of residence

(n = 271,099; male = 37.9%)

Respondents born between 1940 and 1978 (n = 189,640; male = 38.9%)

Complete cases

(n = 169,061; male = 38.7%)

Respondents with missing information on variables

(n = 20,128 ^a^; male = 40.1%)

- PHQ-8 score (n = 18,493; male = 40.5%)
- The number of teeth lost (n = 2,263; male = 35.8%)

a some were duplicated

Respondents with lack of information on age in years

(n = 2,118; male = 26.8%)

Respondents born before 1940 or after 1978

(n = 79,341; male = 35.7%)

Respondents with lack of information on fluoride exposure (n = 451; male = 37.7%)

Respondents who linked with information on fluoride information

(n = 189,189; male = 38.9%)

Respondents in 36 states with Anxiety and Depression module

(n = 317,672; male = 38.0%)

Respondents with lack of information on county of residence (n = 46,573; male = 38.6%)

Respondents to BRFSS in 2006, 2008, and 2010

(n = 1,221,294; male = 37.8%)

- Puerto Rico or Virgin Islands (n = 20,248; male = 35.4%)
- States without Anxiety and Depression module (n = 883,374; male = 37.8%)

**eFigure 3.** Causal effect of tooth loss on a total score of PHQ-8; adjusted for the year of birth dummy, wave of survey dummy, gender, and state of residence; mean, median, and mode of clinically examined number of lost teeth reported in a previous study (*Sekundo C, et al. Patients’ self-reported measures of oral health—A validation study on basis of oral health questions used in a large multi-country survey for populations aged 50+. Gerodontology. 2019;36(2):171-179*) were assigned for the brackets of self-reported 1–5 or 6–27 lost teeth; *P<0.05, **P<0.01, ***P<0.001; confidence intervals for OLS estimation were too narrow to illustrate; Ordinary Least Squares, OLS; the eight-item Patient Health Questionnaire depression scale, PHQ-8

**eFigure 4.** Causal effect of tooth loss on each component of PHQ-8; the eight-item Patient Health Questionnaire depression scale; N = 169,061

**eMethod 1.** PHQ-8 questionnaire in the Behavioral Risk Factor Surveillance System

- Questionnaire in the BRFSS survey (respondents answered the number of days that they experienced the eight symptoms):

Over the last 2 weeks, how many days have you:

- Had little interest or pleasure in doing things?
- Felt down, depressed or hopeless?
- Had trouble falling asleep or staying asleep or sleeping too much?
- Felt tired or had little energy?
- Had a poor appetite or eaten too much? (Variable name change)
- Felt bad about yourself or that you were a failure or had let yourself or your family down?
- Had trouble concentrating on things, such as reading the newspaper or watching the TV?
- Moved or spoken so slowly that other people could have noticed? Or the opposite--being so fidgety or restless that you were moving around a lot more than usual?
- Conversion of response set in the BRFSS (the number of days) to the PHQ-8 original response set (*Kroenke K, et al. The PHQ-8 as a measure of current depression in the general population. J Affect Disord 2009; 114: 163–73*); the scores were summed and used in the present study

| BRFSS response | PHQ-8 original response | Score |
| --- | --- | --- |
| 0–1 day | Not at all | 0 |
| 2–6 days | Several days | 1 |
| 7–11 days | More than half the days | 2 |
| 12–14 days | Nearly every day | 3 |

Abbreviations: BRFSS, Behavioral Risk Factor Surveillance System; PHQ-8, the eight-item Patient Health Questionnaire depression scale

**eMethod 2.** Comparison of the effect size with previous randomized controlled trials (RCTs) on antidepressant drugs

In a systematic review of RCTs (*Cipriani A, et al. Comparative efficacy and acceptability of 21 antidepressant drugs for the acute treatment of adults with major depressive disorder: a systematic review and network meta-analysis. Lancet. 2018;391(10128):1357-1366*), the effect of antidepressant drugs ranges from 1.37 to 2.13 in terms of odds ratio (OR).

In our data, the prevalence of major depression was 10.76% while losing one tooth causally increased the probability of clinical major depression by 0.81 percentage-points. The effect size was converted to OR by the following calculation:

P_control_ = Probability of having major depression in the population = 0.1076

P_treatment_ = Probability of having major depression those who lost one tooth = 0.1076 + 0.0081 = 0.1157

OR of major depression for one tooth loss = (P_treatment_ / 1−P_treatment_) / (P_control_ / 1−P_control_)

= (0.1157 / (1−0.1157)) / (0.1076 / (1−0.1076)) = 1.085

The OR of major depression for those having lost 10 teeth = 1.085^10 = 2.26
